# Assessing a measure for Quality of Life in patients with severe Alopecia Areata: a multicentric Italian study

**DOI:** 10.3389/fpubh.2024.1415334

**Published:** 2024-08-16

**Authors:** Giacomo Caldarola, Giulia Raimondi, Tonia Samela, Lorenzo Pinto, Francesca Pampaloni, Michela Valeria Rita Starace, Laura Diluvio, Federica Dall'Oglio, Emanuele Vagnozzi, Maria Beatrice de Felici del Giudice, Riccardo Balestri, Francesca Ambrogio, Giampiero Girolomoni, Silvia Francesca Riva, Francesco Moro, Laura Atzori, Giuseppe Gallo, Simone Ribero, Oriana Simonetti, Stefania Barruscotti, Valeria Boccaletti, Angelo Valerio Marzano, Luca Bianchi, Giuseppe Micali, Bianca Maria Piraccini, Maria Concetta Fargnoli, Damiano Abeni, Ketty Peris

**Affiliations:** ^1^UOC di Dermatologia, Dipartimento di Scienze Mediche e Chirurgiche, Fondazione Policlinico Universitario A. Gemelli—IRCCS, Rome, Italy; ^2^Dermatologia, Dipartimento di Medicina e Chirurgia Traslazionale, Università Cattolica del Sacro Cuore, Rome, Italy; ^3^Clinical Epidemiology Unit, Istituto Dermopatico dell'Immacolata, IDI-IRCCS, Rome, Italy; ^4^Clinical Psychology Unit, Istituto Dermopatico dell'Immacolata, IDI-IRCCS, Rome, Italy; ^5^Dermatology Unit, IRCCS Azienda Ospedaliero-Universitaria di Bologna, Bologna, Italy; ^6^Department of Medical and Surgical Sciences University of Bologna, Bologna, Italy; ^7^Dermatology Unit, Fondazione Policlinico di Tor Vergata, Tor Vergata University of Rome, Rome, Italy; ^8^Dermatology Clinic, University of Catania, Catania, Italy; ^9^Department of Biotechnological and Applied Clinical Sciences, University of L'Aquila, L'Aquila, Italy; ^10^UOC Dermatologia, Dipartimento di Medicina e Chirurgia, Università di Parma, Parma, Italy; ^11^Division of Dermatology, “U.O. Multizonale APSS”, Trento, Italy; ^12^Section of Dermatology and Venereology, Department of Precision and Regenerative Medicine and Ionian Area (DiMePRe-J), University of Bari “Aldo Moro”, Bari, Italy; ^13^Dipartimento di Medicina, Sezione di Dermatologia, Università di Verona, Verona, Italy; ^14^Department of Health Sciences (DISSAL), Section of Dermatology, University of Genoa, Genoa, Italy; ^15^Department of Dermatology, Ospedale Policlinico San Martino IRCCS, Genoa, Italy; ^16^Dermatology Unit, IDI-IRCSS, Rome, Italy; ^17^Dermatology Unit, Department Medical Sciences and Public Health, University of Cagliari, Cagliari, Italy; ^18^Dermatology, Department of Medical Sciences, University of Turin, Turin, Italy; ^19^Clinica Dermatologica—Dipartimento di Scienze Cliniche e Molecolari Università Politecnica delle Marche, Azienda Ospedaliero-Universitaria Ospedali Riuniti di Ancona, Ancona, Italy; ^20^Dermatology Clinic, Fondazione IRCCS Policlinico San Matteo, Pavia, Italy; ^21^Department of Clinical-Surgical, Diagnostic and Pediatric Sciences, University of Pavia, Pavia, Italy; ^22^Clinica Dermatologica, Università degli Studi di Brescia, Brescia, Italy; ^23^Dermatology Unit, Fondazione IRCCS Ca' Granda Ospedale Maggiore Policlinico, Milan, Italy; ^24^Department of Pathophysiology and Transplantation, Università degli Studi di Milano, Milan, Italy

**Keywords:** Alopecia Areata (AA), Quality of Life, Bayesian confirmatory factor analysis, psychodermatology, patients reported outcomes

## Abstract

**Objective:**

The prevalence of anxiety and depression in patients diagnosed with Alopecia Areata (AA) is very high and this significant burden of psychological symptoms threatens the Health-Related Quality of Life (HRQoL) of affected patients. Indeed, AA often does not produce significant physical symptoms, but it nonetheless disrupts many areas of mental health. Clinical assessment of disease severity may not reliably predict patient's HRQoL, nor may it predict the patient's perception of illness. For this reason, considerable effort has been made to apply and develop measures that consider patient's perception and assess the HRQoL of individuals affected by AA. The aim of this multicentric study was to provide the Italian version of the Skindex-16AA and to evaluate its psychometric properties in a clinical sample of consecutive patients with moderate-to-severe AA.

**Methods:**

This is a longitudinal, multicenter, observational study. Patients returned for follow-up visits at 4-, 12-, and 24-weeks. The analyses of the current work aimed to confirm the factorial structure of the Skindex-16AA. In the case of non-fit, an alternative structure for the model was proposed, using an Exploratory Graph Analysis and the Bayesian approach.

**Results:**

The sample was composed of 106 patients with AA. Alopecia Universalis was the most frequently diagnosed type of alopecia at all time points. The analyses on the Skindex-16AA revealed that a two-factor structure with eight items fit the data best (Bayesian Posterior Predictive Checking using 95% Confidence Interval for the Difference Between the Observed and the Replicated Chi-Square values = −6.246/56.395, Posterior Predictive *P*-value = 0.06), and reported satisfactory psychometric properties (i.e., internal consistency and convergent validity).

**Conclusion:**

The Skindex-8AA demonstrated optimal psychometric properties (i.e., convergent and construct validity, and test-retest reliability) measured in a sample of patients with AA, that may suggest that it is an appropriate tool to measure the HRQoL in AA patients. However, further studies are needed in order to confirm and tested other psychometric features of this tool.

## 1 Introduction

Alopecia Areata (AA) is an autoimmune disease with a chronic-recurrent course and a multifactorial pathogenesis, characterized by heterogeneous patterns of hair loss. It represents the second most common form of non-scarring alopecia after androgenetic alopecia, with an estimated lifetime prevalence of 0.10% in the general population worldwide (1); all ages are susceptible; however children and young adults seem to be most commonly more affected (2).

The etiopathogenesis of AA is not yet fully understood, although there is consensus in the literature regarding the involvement of predisposing genetic factors and environmental triggers in determining the onset of the disease (3). Loss of immune privilege by the hair follicle with subsequent activation of CD8 positive T-Lymphocytes seems to be the driver of the disease (4, 5). AA may be associated with various systemic diseases including atopic diseases, thyroid disorders, vitiligo, psoriasis, celiac disease, ulcerative rettocolitis and rheumatoid arthritis (2).

Clinically, AA is characterized by sudden patchy hair loss, involving the scalp or other parts of the body without signs of inflammation or scarring. The most common clinical variants of AA are patchy alopecia, alopecia totalis (AT), alopecia universalis (AU), ophiasis, sisaipho and Marie Antoinette and Thomas More syndrome (3). There are several classification systems for clinical severity of alopecia, however, the Severity of Alopecia Tool (SALT), proposed by Olsen et al., appears to be the most widely used instrument (6). The scoring of the SALT is based on clinician observation and consists of assessing the percentage of scalp area affected by alopecia (i.e., dividing the scalp area into four parts: back 24%, top 40%, both sides 18% of the head). A percentage of involved area >50% results in a score classified as “severe AA” (6).

The prevalence of anxiety and depression in children, as well as in adults, diagnosed with AA is very high (7, 8); furthermore, stress and anxiety can also be considered as triggers of the disease (9). This significant burden of psychological symptoms threatens the Health-Related Quality of Life (HRQoL) of patients. Indeed, AA often does not produce significant physical symptoms, but it appears to disrupt many areas of mental health (10).

Over the years, several disease-specific instruments have been validated to measure the clinical severity of the disease, however, clinical assessment of disease severity does not reliably predict the patient's HRQoL, nor does it predict the patient's perception of illness (11, 12). For this reason, considerable effort has been made to apply and develop measures that consider the patient's perception and assess the HRQoL of individuals affected by AA (13). In fact, a position statement of the European Academy of Dermatology and Venereology Task Force on Quality of Life and Patient Oriented Outcomes explicitly stated that the development and validation of AA-specific instruments would be extremely useful to encourage (14). Several generic and dermatology-specific HRQOL instruments have been used, such as Alopecia Areata Quality of Life Index (15), Alopecia Areata Quality of Life (16), or Alopecia Areata Symptom Impact Scale (17), but they are all lacking of important aspects for their construct validity (13). Moreover, no validation studies for the questionnaires have been proposed on samples of Italian patients. Among different questionnaires proposed, only the Skindex in its different forms (18–20) has been proved to possess satisfactory validity across several, yet different dermatological conditions.

Specifically, the Skindex-16 (13, 21, 22) consists of 16 items that assess the impact of skin disease on three domains: symptoms, emotions, and functioning. Each item is scored on a 7-point Likert scale, ranging from 0 (never bothered) to 6 (always bothered). The total score ranges from 0 to 96, with higher scores indicating greater impairment in quality of life. The Skindex-16 has demonstrated good reliability and validity across various dermatological conditions and has been translated and validated in multiple languages. It serves as a tool for assessing the impact of skin diseases on patients' quality of life in both clinical and research settings (18). The Skindex-16AA was proposed by Gelhorn et al. (20) with slight wording changes respect to Skindex-16 to refer to scalp and AA. This form of the questionnaire was used in the clinical trials BRAVE-AA1 and -AA2, which evaluated the efficacy and safety of baricitinib in AA patients (23). This is a Janus kinase (JAK) inhibitor that modulates immune responses by selectively inhibiting JAK1 and JAK2 enzymes. It is administered orally and it has shown great efficacy in the treatment of AA, being the first drug approved for this condition (24).

Therefore, the aim of the current multicentric study was to provide the Italian adaptation of the Skindex-16AA and to evaluate its psychometric properties (i.e., convergent validity and test-retest reliability) in a real-life clinical sample of consecutive patients with moderate-to-severe AA, among whom a subgroup was treated with baricitinib. Specifically, the main aim was to evaluate the dimensionality of the Skindex-16AA (18) in an Italian clinical sample of patients with AA, and, in case of non-adequacy of the model, to provide an alternative factor structure and/or revision.

## 2 Materials and methods

### 2.1 Study participants

Patients were recruited from different Italian centers^1^, from the January to October 2023. We enrolled patients aged >18 years, diagnosed with a severe AA (defined as SALT score >50%) by a dermatologist. Baricitinib treatment (4 mm/die, for at least 1 year) was administered to patients unresponsive to conventional systemic treatments (i.e., oral cyclosporine; and topical, oral, intramuscular and intravenous steroids). Participants voluntarily agreed to participate in the study and signed informed consent.

Patients returned for follow-up visits after 4, 12, and 24-weeks (see Table 1 for descriptive statistics both at baseline and follow-up).

**Table 1:** Descriptive statistics of the sample.

**Variables**	**Baseline (*N* = 106)**	**4-weeks follow-up (*N* = 77)**	**12-weeks follow-up (*N* = 38)**	**24-weeks follow-up (*N* = 35)**
**Sex** ***N*****/%**				
Women	77 (72.64%)	56 (72.72%)	28 (73.68%)	27 (77.14%)
Men	29 (27.35%)	21 (27.27%)	10 (26.32%)	8 (22.85%)
Age *M*/(SD)	40.20 (±12.56)	40.80 (±12.28)	41.00 (±12.16)	41.08 (±12.08)
Age diagnosis	27.72 (±15.25)	27.85 (±15.56)	28.07 (±15.25)	29.45 (±17.22)
**Type of AA** ***N*****/%**				
Alopecia totalis	26 (24.52%)	19 (24.67%)	10 (26.31%)	10 (28.57%)
Alopecia universalis	61 (57.54%)	47 (61.03%)	25 (65.78%)	21 (60%)
Patchy alopecia (≥50%)	19 (17.92%)	11 (14.28%)	3 (7.89%)	4 (11.42%)
Systemic treatment *N*/%	15 (14.15%)	9 (11.68%)	4 (10.52%)	3 (8.57%)
Family history for AA *N*/%	25 (23.58%)	20 (25.97%)	9 (23.68%)	11 (31.42%)
SALT *M*/(SD)	90.79 (±18.85)	84.87 (±22.57)	61.46 (±36.48)	39.64 (±40.09)
HADS-depression *M*/(SD)	8.81 (±11.25)	6.23 (±5.88)	4.14 (±3.44)	6.92 (±6.43)
HADS-anxiety *M*/(SD)	7.83 (±6.08)	7.50 (±6.21)	6.25 (±4.51)	5.75 (±5.34)

*N*, number count; %, percentage; M, mean; SD, standard deviation; AA, Alopecia Areata; SALT, severity of Alopecia tool; HADS, Hospital Anxiety and Depression Scale.

The study was approved by the Institutional Ethical Committee of IDI-IRCCS (i.e., Istituto Dermopatico dell'Immacolata-Istituto di Ricovero e Cura a Carattere Scientifico) (protocol number: 739/1).

### 2.2 Measures

At both baseline and follow-up evaluations, socio-demographic (i.e., age and sex) and clinical variables (e.g., severity of AA, age of diagnosis) were registered (see Table 1), and patients completed the Italian version of the following questionnaire, Skindex-16AA (9, 14) and the Hospital Anxiety and Depression Scale (HADS) (15).

The Skindex-16AA is a 16-item self-report questionnaire assessing measuring the level of quality of life impairment caused by a AA (20). It measures 3 domains, Symptoms (items from #1 to #4), Emotion (items from #5 to #11) and Functioning (items from #12 to #16), and higher score on all dimensions reflect greater impact of AA. The Italian version of the Skindex-16AA was obtained through the conventional back-translation procedure. The content validity of the item has been assessed by one of the Authors (GC) specialized in the treatment of patients with AA, as well as a group of patients (*N* = 5), who were asked if there were difficulties in the understanding of the items. None of the patients declares issues in the comprehension of the items.

The HADS (25) is a 14-item self-report questionnaire measuring anxiety (e.g., “*Worrying thoughts go through my mind*”) and depressive (e.g., “*I feel as if I am slowed down*”) symptoms. It is commonly used in both clinical and non-clinical samples (26). Both subscales are composed of seven items each, which are rated on a four-point scale (i.e., 0–3), with higher scores indicating more severe depressive and anxiety symptoms. The psychometric properties of the HADS demonstrated satisfactory results (27). Specifically, for the Italian population (28), the HADS demonstrated satisfactory construct validity and internal consistency.

### 2.3 Statistical analyses

All analyses were performed with JASP (version 0.17.1), Mplus (version 8) (29), and EGAnet (version 1.2.3) for R studio (version 4.2.3). Exploratory Graph Analysis (EGA) (30) and Bayesian confirmatory factor analysis were used to investigate dimensionality of the scale.

Firstly, a Bayesian Confirmatory Factor Analysis (BCFA) was conducted on the Skindex-16AA in order to test the adequacy of the model. In case of non-optimal fit, a more refined exploratory approach, through the Exploratory Graph Analysis (EGA), was used in order to provide an adequate structure of the questionnaire, by removing problematics items.

EGA is a statistical approach that can identify dimensionality (i.e., communities) in multivariate data using network models. In network models, variables are considered as nodes and they are connected by edges that indicate the strength of the association between these variables (i.e., partial correlation coefficients) (31). In the current analyses, items of the Skindex-16AA were the nodes of the model and the graphical least absolute shrinkage and selection operator (GLASSO) estimation method was used to estimate the model's parameters. The hyperparameter γ was set to 0.5, and the Walktrap community detection algorithm was used to detect the number of dimensions (31). Moreover, a bootstrap approach with 1,000 iterations was used to assess the stability of the solution (30). In the first step of the analyses, EGA assesses consistency of the number and membership of the communities (i.e., how often items are included in the same dimensions). Adequate solutions are obtained when the number of dimensions is stable and when the stability of the items is ≥0.70. Item stability refers to how consistently a node is connected to the others. When unstable items are present in a network they are removed, and all the analyses are performed again until the number and composition of dimensions are stable.

As final step, the model retrieved from the EGA was evaluated through a Bayesian confirmatory factor analysis (BCFA) with a Markov chain Monte Carlo (MCMC) algorithm. We used GIBBS sampling algorithm and 100,000 post burn-in iterations (32). Bayesian analysis is the most appropriate approach when dealing with small sample size (33). Moreover, in the Bayesian approach all parameters are treated as uncertain (i.e., and not as fixed effect like in the frequentist approach), and models can be tested using different prior distribution, which can affect the precision of parameters estimation. In other words, the smaller the prior variance, the higher the precision of parameters estimation (34). Weak informative priors [*N* (0, 1.0)] were used for the hypothesized factor loadings. Sensitivity of the model to priors was inspected comparing the hypothesized model with two competing models which increasingly favor the null hypothesis for factor loadings [*N* (0, 0.25) and *N* (0, 0.10)]. The models fit was evaluated using the Bayesian Posterior Predictive Checking (PPC) and the Posterior Predictive *P*-value (PPP) (35). The fit of the model was based on the PPC confidence interval crossing the zero and PPP > 0.05.

Indices of internal consistency were reported as Cronbach's α and McDonald's ω with their 95% credible intervals (36). As measure of convergent validity, we reported Pearson correlation coefficients with the HADS and also assessed the test-retest reliability of the revised version of the Skindex-16AA at 4, 12, and 24 weeks follow-up. In addition, after obtaining the revised version, we tested the correlation coefficients between the original and the revised version of the Skindex-16AA.

Finally, responsiveness of the revised version of the Skindex-16AA was calculated with *t*-test analysis for paired samples in order to assess the magnitude of the difference in the QoL score between baseline and 24-weeks follow-up. Effect size of 0.20 is considered small, of 0.50 moderate and of 0.80 large (37).

## 3 Results

### 3.1 Descriptive statistics

At baseline, the sample was composed of 106 patients (72.64% women), with a mean age of 40.20 years (SD = 12.56); at 4-weeks follow-up of 77 patients (72.72% women), with a mean age of 40.80 (SD = 12.28); at 12-weeks follow-up, of 38 patients (73.68% women), with a mean age of 41 years (SD = 12.16); and finally, at 24-weeks follow-up of 35 patients (77.14% women), with a mean age of 41.08 (SD = 12.08). Alopecia Universalis was the most diagnosed type of alopecia in the sample at the baseline (57.54%), compared to the Alopecia Totalis (24.52%) and Patchy Alopecia (17.92%). The SALT mean score decreased during the study period, starting from a mean score of 90.79 (SD = 18.85) at the baseline, of 84.87 (SD = 22.57) at 4-weeks follow-up, of 61.46 (SD = 36.48) at 12-weeks follow-up, to 39.64 (SD = 40.09) at 24-weeks follow-up, which reflects an improvement in the extent and severity of hair loss in our sample.

### 3.2 Exploratory graph analysis

The original model of the Skindex with 16 items (i.e., Skindex-16AA) (18) was not confirmed with the BCFA (Bayesian Posterior Predictive Checking using 95% Confidence Interval for the Difference Between the Observed and the Replicated Chi-Square values = 9.144/115.372; Posterior Predictive *P*-Value = 0.01), and although all items loaded significantly (< 0.001) on their hypothesized dimensions, this indicated the presence of problematic items.

**Figure 1:**
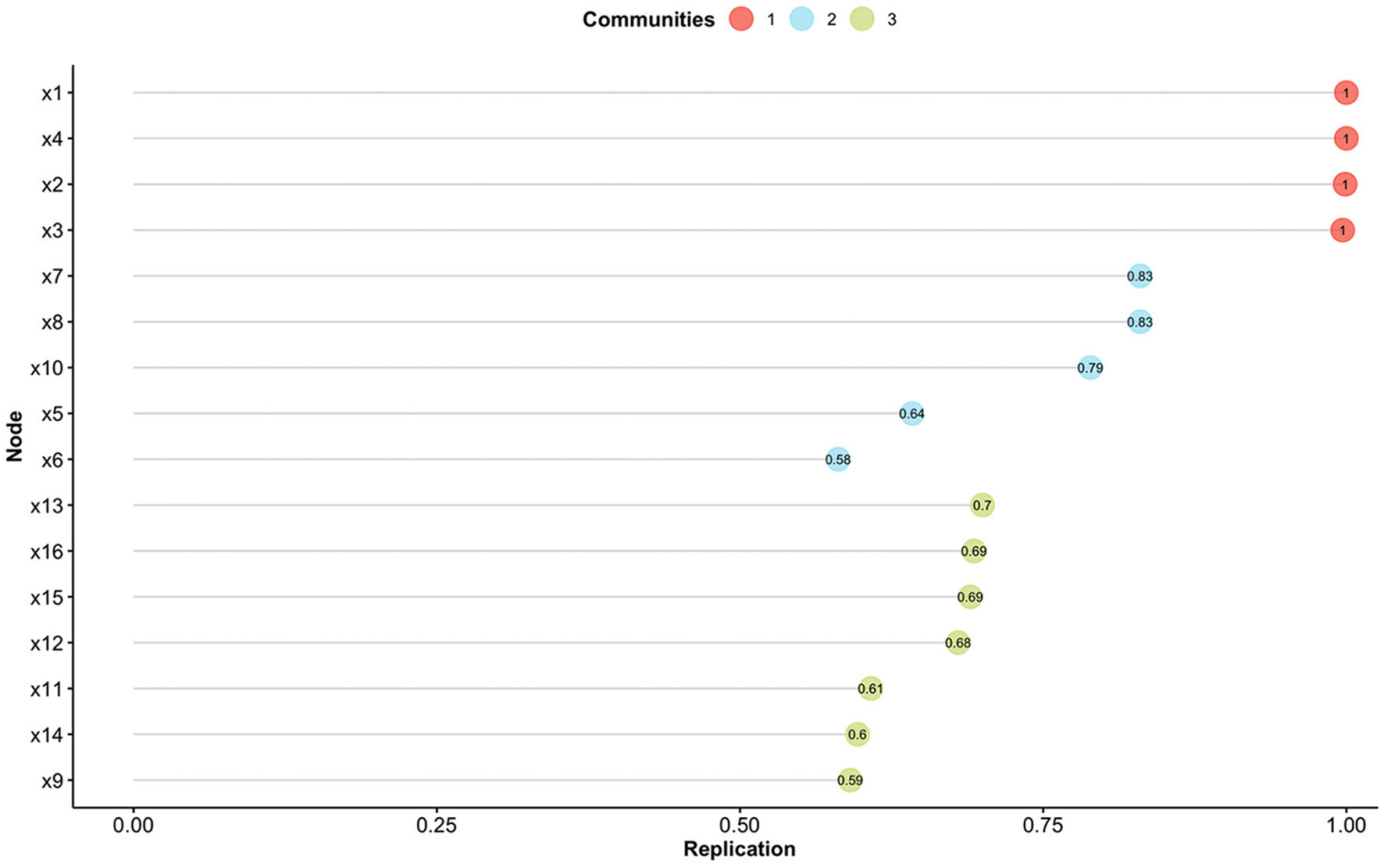
Item stability analysis for the Exploratory Graph Analysis. Different colors indicate the different communities found with the Exploratory Graph Analysis. Nodes (i.e., items) with a value < 0.70 are not considered stable.

Therefore, the model of the Skindex-16AA was further explored with the EGA, which initially suggested a three-factor solution. Stability analysis revealed the presence of eight items which were unstable (item's stability < 0.70; items #5, #6, #9, #11, #12, #14, #15, #16) (see Figure 1). Another EGA analysis was conducted with the remaining 8 items, which suggested the presence of two factors, with four items for each dimension. We named this revised version Skindex-8AA, and all its items reported a high stability (=1.00).

### 3.3 Bayesian confirmatory factor analysis

The BCFA was performed on the model suggested by the EGA, Skindex-8AA, and reported a good fit to the data (Bayesian Posterior Predictive Checking using 95% Confidence Interval for the Difference Between the Observed and the Replicated Chi-Square values = −15.287/41.715; Posterior Predictive *P*-Value = 0.18), with all items loaded significantly (< 0.001) and >0.40 on their hypothesized dimensions (see Table 2; see Supplementary material for the Italian version of the Skindex-8AA).

**Table 2:** Bayesian confirmatory factor analysis of the Skindex-8AA.

	**Symptoms**	**Social-emotional**	**95% CI**
Item 1 (itching)	0.790		0.684–0.866
Item 2 (burning/stinging)	0.917		0.860–0.953
Item 3 (hurting)	0.796		0.684–0.873
Item 4 (irritation)	0.852		0.764–0.911
Item 7 (appearance)		0.881	0.805–0.930
Item 8 (frustration)		0.909	0.846–0.946
Item 10 (ANNOYED)		0.845	0.759–0.904
Item 13 (interactions)		0.852	0.768–0.911
**Mean scores (SD)**			
Baseline	6.50 (5.23)	15.69 (7.14)	
4-weeks follow-up	5.62 (4.08)	16.21 (7.11)	
12-weekes follow-up	3.57 (2.21)	11.68 (7.78)	
24-weeks follow-up	5.71 (3.67)	11.05 (7.24)	

95% CI, 95% credibility intervals; SD, standard deviation.

Sensitivity analysis to priors suggested an effect of priors to parameters estimates. When using informative priors which increasingly favor the null hypothesis for factor loadings [*N* (0, 0.25) and *N* (0, 0.10)], the models indicated the same adequate fit for the moderate informative prior (Bayesian Posterior Predictive Checking using 95% Confidence Interval for the Difference Between the Observed and the Replicated Chi-Square values = −6.246/56.395, Posterior Predictive *P*-Value = 0.06), but a significant model fit for the strong informative prior (Bayesian Posterior Predictive Checking using 95% Confidence Interval for the Difference Between the Observed and the Replicated Chi-Square values = 18.103/96.140, Posterior Predictive *P*-Value = 0.001). Lastly, changes in estimated factor loadings were all below 10% with both priors.

### 3.4 Psychometric properties of the Skindex-8AA

The two factors of the Skindex-8AA were named Symptoms and Social-Emotional, respectively. The Symptoms factor had McDonald's ω posterior mean of 0.866 (95% CI = 0.824–0.903, posterior probability 0.70 < ω < 0.90 = 0.964; Cronbach α = 0.867, 95% CI = 0.827–0.909, posterior probability 0.70 < α < 0.90 = 0.953) and the Social-Emotional factor had McDonald's ω posterior mean of 0.915 (95% CI = 0.888–0.939, posterior probability 0.70 < ω < 0.90 = 0.134; Cronbach α = 0.919, 95% CI = 0.896–0.945, posterior probability 0.70 < α < 0.90 = 0.076), ensuring the construct validity of the Skindex-8AA.

Both factors demonstrated satisfactory and significant convergent validity with the HADS subscales (i.e., depression and anxiety) (Symptoms with HADS depression: *r* = 0.38 *p* < 0.01; Symptoms with HADS anxiety: *r* = 0.35, *p* < 0.01; Social-Emotional with HADS depression: *r* = 0.30, *p* < 0.05; Social-Emotional with HADS anxiety: *r* = 0.32, *p* < 0.01). Finally, Skindex-8AA reported adequate and significant test-retest reliability, at 4, 12, and 24 weeks follow-up (see Table 3).

**Table 3:** Skindex-8AA test-retest reliability.

**Baseline**	**4 weeks (*****N*** = **79)**		**12 weeks (*****N*** = **40)**		**24 weeks (*****N*** = **36)**	
	**Symptoms**	**Social-emotional**	**Symptoms**	**Social-emotional**	**Symptoms**	**Social-emotional**
Symptoms	0.68^**^	0.43^**^	0.59^**^	0.31^*^	0.61^**^	0.65^**^
Social-emotional	0.34^**^	1^**^	0.40^*^	0.73^**^	0.37^*^	0.77^**^

^**^p < 0.01; ^*^p < 0.05.

In addition, the correlations between the Skindex-16AA and the newly proposed Skindex-8AA were found to be very high. The Symptoms subscales had an *r* = 1; the Social-Emotional subscale of the Skindex-8AA had an *r* = 0.95 with the Emotional subscale of the Skindex-16AA and an *r* = 0.86 with the Functioning subscale of the Skindex-16AA. These results indicate that the information provided by the new version proposed (i.e., Skindex-8AA) is practically superimposable to the one of the Skindex-16AA.

Finally, the responsiveness analysis demonstrated that the Social-Emotional subscale of the Skindex-8AA was responsive to change, but not the Symptoms subscale. Mean Skindex-8AA score at baseline was of 16.37 (SD = 7.16) and of 11.05 (SD = 7.24) at 24-weeks follow-up, with an effect size of 1.08 (Cohen's *D*) (*p* < 0.001), indicating a large significant change in score from baseline to 24-weeks follow-up.

## 4 Discussion

The main aim of the current study was to provide the Italian adaptation of the Skinde-16AA and study its psychometric properties, in a group of Italian patients with severe AA. However, in case of non-adequacy of the model of the Skindex-16AA, we aimed at proposing an alternative factor structure and/or revision.

The factor analysis did not confirm the original factor structure of the Skindex-16AA (18) due to the presence of problematic items, which were removed from the analyses. The final structure of the questionnaire resulted in two dimensions, that were named Symptoms and Socio-Emotional, composed by four item each, which we named Skindex-8AA. Although the Posterior Predictive *P*-Value became significant with a strong informative prior, the changes in factor loadings were < 10% compared to the model tested with the weaker priors, indicating that the interpretation of the model remained stable with different priors. Moreover, it is important to have short and reliable measure because shorter questionnaires are easier to administer, less time-consuming, and allow for the administration of multiple measures (e.g., for epidemiological studies), thereby reducing the burden on patients.

In our sample, in the first 24 weeks of treatment, our patients experienced a halving of the SALT score, in association with a mild improvement in the mean scores of the anxiety and depression domains of HADS.

Having a specific tool assessing quality of life in patients with AA is of utmost important, since AA significantly affects self-image, and psychosocial factors related to this condition often negatively impact patient QoL, especially in women, as found in the current study and confirmed by literature (29). Moreover, AA is associated with a significant mental health burden including anxiety, depression, suicidal ideation and/or behavior and high rates of psychiatric hospitalizations (21, 38). Whether the psychiatric comorbidity such as anxiety or depression leads to AA or vice versa is still poorly understood. Major depressive disorder was found to increase the risk of developing this condition by 90%, and AA, conversely, was found to increase the risk of subsequently developing major depressive disorder by 34% in adult population (8). Furthermore, the lack of control on the progression and relapses of AA puts patients on a higher risk of feel sad and helpless about their diagnosis, leading to anxiety, worry, fearful, and distress about disease recurrences (10).

The association between hair loss and mood disorders, however, was not demonstrated in pediatric samples. While earlier studies suggested that children may be less prone to developing depression and anxiety symptomatology until they reach an older age, when peers relationships become more salient (39), recent research indicates otherwise. Systematic reviews and studies, such as those by Toussi et al. (21), Vélez-Muñiz et al. (40), and Sinclair (41), have addressed the QoL and the presence of depression, anxiety, and suicide risk in pediatric populations with AA. These studies highlight that children with AA may indeed experience significant psychological distress, challenging the notion that younger children are largely unaffected by their condition in terms of mood disorders.

The psychosocial concern about others' judgment and fear of rejection seem to play a significant role; particularly hair loss in areas that were visible to others were most psychosocially burdensome to patients. Qualitative studies, in fact, suggested that people who suffered from AA frequently reported feelings of insecurity, inadequacy, and lack of self-consciousness due to the changes in their appearance that could impact on the self-identity and self-perception (42). Indeed, the incidence of body dysmorphic disorder is about 13 times higher in patients with AA than in general dermatology patients (43). All these psychological concerns could interfere in the way that patients may rate their condition, so patients tend to rate their AA as more severe and more invariable than dermatologists assessing it by SALT (44). This may explain why depression and anxiety symptoms tend to decrease slower than the SALT score during the treatment, as seen in the quality-of-life scores in psychosocial and symptoms domains.

The Skindex-8AA demonstrated to have an optimal test-retest reliability, over a wide time-frame, which indicates that its factor structure is stable over time.

Overall, specific instruments are usually more responsive than generic tools (45), and may better detect specific characteristics of the patient's illness experience, so it might be useful for clinicians and researchers to consider the use of these questionnaires when evaluating or studying Alopecia Areata.

In conclusion, the Skindex-8AA demonstrated satisfactory psychometric properties (i.e., convergent and construct validity, and test-retest reliability) as assessed in a sample of patients with AA. Future studies are needed to confirm its factor structure on a wider sample, with a more balanced number of women and men, and to assess its congruent validity with another measure of Health-related quality of life tool.

## Data availability statement

The raw data supporting the conclusions of this article will be made available by the authors, upon reasonable request.

## Ethics statement

The studies involving humans were approved by the Institutional Ethical Committee of IDIIRCCS (protocol number: 739/1). The studies were conducted in accordance with the local legislation and institutional requirements. The participants provided their written informed consent to participate in this study.

## Author contributions

GC: Conceptualization, Writing – original draft, Writing – review & editing. GR: Conceptualization, Formal analysis, Methodology, Visualization, Writing – original draft, Writing – review & editing. TS: Visualization, Writing – original draft, Writing – review & editing, Validation. LP: Writing – original draft, Writing – review & editing. FP: Writing – original draft, Writing – review & editing. MS: Writing – original draft, Writing – review & editing. LD: Writing – original draft, Writing – review & editing, Conceptualization, Funding acquisition. FD'O: Writing – original draft, Writing – review & editing. EV: Writing – original draft, Writing – review & editing. MFG: Writing – original draft, Writing – review & editing. RB: Writing – original draft, Writing – review & editing. FA: Writing – original draft, Writing – review & editing. GGi: Writing – original draft, Writing – review & editing. SFR: Writing – original draft, Writing – review & editing. FM: Writing – original draft, Writing – review & editing. LA: Writing – original draft, Writing – review & editing. GGa: Writing – original draft, Writing – review & editing. SR: Writing – original draft, Writing – review & editing. OS: Writing – original draft, Writing – review & editing. SB: Writing – original draft, Writing – review & editing. VB: Writing – original draft, Writing – review & editing. AM: Writing – original draft, Writing – review & editing. LB: Writing – original draft, Writing – review & editing. GM: Writing – original draft, Writing – review & editing. BP: Writing – original draft, Writing – review & editing. MCF: Writing – original draft, Writing – review & editing. DA: Supervision, Writing – original draft, Writing – review & editing. KP: Writing – original draft, Writing – review & editing, Supervision, Validation. Italian Study Group on Cutaneous Adnexal Disease of the Italian Society of Dermatology and Sexual Transmitted diseases (SIDeMaST): Writing – original draft, Writing – review & editing.

## Group members of the Italian Study Group on Cutaneous Adnexal Disease of the Italian Society of Dermatology and Sexual Transmitted diseases (SIDeMaST)

### Consortium members

Ersilia Tolino^1^, Gianluca Landucci^2^, Maria Cristina Acri^3^, Alfredo Rossi^3^, Giovanni Pellacani^3^, Francesco Lacarruba^4^, Francesco Bellinato^5^, Francesca Prignano^6^, Carlo Tomasini^7,8^, Alessandro Fraghì^9^, Silvia Mariel Ferrucci^10^, Maria Alessandra Mattioli^11^, Mauro Barbareschi^11^, Lorenzo Rocca^11^, Tommaso Ioris^12^, Raffaele Dante Caposiena^13^, Iris Zalaudek^13^, Chiara Caponio^14^, Pietro Rubegni^14^, Elisa Cinotti^14^, Emanuele Trovato^14^, Marco Romanelli^15^, Valentina Dini^15^, Flavia Manzo Margiotta^15^, Claudio Feliciani^16^, Lorenzo Ala^17^, Silvia Sanna^17^, Serena Lembo^18^, Annunziata Raimondo^18^, Michela Magnano^19^.

### Consortium affiliations

^1^ Dermatology Unit “Daniele Innocenzi”, “A. Fiorini” Hospital, Via Firenze, 1, Terracina, Italy.

^2^ Department of Health Sciences, University of Eastern Piedmont, Novara, Italy.

^3^ Dipartimento di Scienze Cliniche Internistiche Anestesiologiche e Cardiovascolari. U.O.C. di Dermatologia, Facoltà di Medicina e Odontoiatria, La Sapienza Università di Roma Viale del Policlinico 155 Roma.

^4^ Dermatology Clinic, University of Catania, Italy.

^5^ Dipartimento di Medicina, Sezione di Dermatologia, Università di Verona.

^6^ Department of Health Sciences, Section of Dermatology, University of Florence, Firenze Italy.

^7^ Dermatology Clinic, Fondazione IRCCS Policlinico San Matteo, Pavia, Italy.

^8^ Department of Clinical-Surgical, Diagnostic and Pediatric Sciences, University of Pavia, Pavia, Italy.

^9^ Clinica Dermatologica, Università degli Studi di Brescia.

^10^ Dermatology Unit, Fondazione IRCCS Ca' Granda Ospedale Maggiore Policlinico, Milan, Italy.

^11^ Department of Pathophysiology and Transplantation, Università degli Studi di Milano, Milan, Italy.

^12^ Division of Dermatology, Trento Hospital, Trento, Italy.

^13^ Dermatology Clinic, Maggiore Hospital, University of Trieste, Trieste, Italy.

^14^ Department of Medical, Surgical and Neurological Science, Dermatology Section, University of Siena, S. Maria alle Scotte Hospital, Siena, Italy.

^15^ Unità di Dermatologia, Dipartimento di Medicina Clinica e Sperimentale, Università di Pisa, Pisa, Italy.

^16^ Unit of Dermatology, Department of Medicine and Surgery, University of Parma, Parma, Italy.

^17^ Dermatology Unit, Department Medical Sciences and Public Health, University of Cagliari, Italy.

^18^ Universita' Di Salerno, Dipartimento Di Medicina, Chirurgia E Odontoiatria “Scuola Medica Salernitana”.

^19^ Unit of Dermatology, Versilia Hospital, Lido di Camaiore, Lucca, Italy.

### Consortium contributions

The Italian Study Group on Cutaneous Adnexal Disease of the Italian Society of Dermatology and Sexual Transmitted diseases (SIDeMaST) contributed to: Writing – original draft, Writing – review & editing.

## Footnotes

^1^Italian centers: Scuola di Dermatologia e Venereologia di Bologna; Policlinico Umberto I, Roma; AOUI Verona; Ospedale San Martino, Genova; Ospedale Maggiore della carità di Novara; Unità di Dermatologia Policlinico di Roma Tor Vergata; Clinica Dermatologica di Milano; Policlinico A. Gemelli, Roma; Azienda Ospedaliero-Universitaria Ospedali Riuniti di Ancona; Unità dermatologica di Pisa; Università di Torino; Fondazione IRCCS Policlinico San Matteo, Pavia; Università di Parma; Ospedale Palagi, Firenze; Ospedale dell'Aquila; Ospedale Universitario di Siena; Università degli studi di Napoli Federico II; U.O.C. Dermatologia G. Rodolico, Catania; Azienda Sanitaria Universitaria Giuliano Isontina (ASUGI), Trieste; Ospedale di Vicenza; IRCCS Ospedale San Raffaele di Milano; Ospedale di Trento; Policlinico di Bari Ospedale Giovanni XXIII; Spedali civili Brescia; Università di Salerno, A.O.U San Giovanni di Dio e Ruggi; Ospedale Versilia.
