# Supplementary material for: Assessing a measure for Quality of Life in patients with severe Alopecia Areata: a multicentric Italian study
Source: Front Public Health. 2024 Aug 16;12:1415334. doi: 10.3389/fpubh.2024.1415334 (PMC11363427; doi:10.3389/fpubh.2024.1415334)
Supplement: Supplementary file 1 [file Table_1.DOCX]

**SKINDEX 8 – Alopecia Areata**

*“ALOPECIA” È IL TERMINE MEDICO CHE INDICA LA CADUTA DEI CAPELLI. QUESTE DOMANDE MISURANO QUANTO LA TUA ALOPECIA HA INFLUITO SULLA TUA VITA DURANTE L'ULTIMA SETTIMANA.*

**Rispondi alle seguenti domande inserendo un valore da 1 a 6 (1=MAI; 6=SEMPRE).**

**Durante l’ultima settimana quanto sei stato infastidito da:**

| 1. Il prurito al cuoio capelluto | 0 | 1 | 2 | 3 | 4 | 5 | 6 |
| --- | --- | --- | --- | --- | --- | --- | --- |
| 2. Il bruciore al cuoio capelluto | 0 | 1 | 2 | 3 | 4 | 5 | 6 |
| 3. Il dolore al cuoio capelluto | 0 | 1 | 2 | 3 | 4 | 5 | 6 |
| 4. L’irritazione al cuoio capelluto | 0 | 1 | 2 | 3 | 4 | 5 | 6 |
| 5. L’aspetto estetico dell’alopecia | 0 | 1 | 2 | 3 | 4 | 5 | 6 |
| 6. La frustrazione per l’alopecia | 0 | 1 | 2 | 3 | 4 | 5 | 6 |
| 7. L’essere irritato per l’alopecia | 0 | 1 | 2 | 3 | 4 | 5 | 6 |
| 8. L’impatto della alopecia sul desiderio di stare con gli altri | 0 | 1 | 2 | 3 | 4 | 5 | 6 |
